# Supplementary material for: Gut microbial composition is altered in sarcopenia: A systematic review and meta-analysis of clinical studies
Source: PLoS One. 2024 Aug 6;19(8):e0308360. doi: 10.1371/journal.pone.0308360 (PMC11302912; doi:10.1371/journal.pone.0308360)
Supplement: S2 Table — (DOCX) [file pone.0308360.s002.docx]

**S2 Table.** Search Strategy and Search Results.

| **S2 Table.** Search Strategy and Search Results | | |
| --- | --- | --- |
| **A** PubMed Search | | |
| Entry | PubMed Search Strategy | Results |
| 1 | ((gut) OR (gastrointestinal) OR (intestinal) OR (fecal) OR (stool)) AND ((microbiome) OR (microbiota) OR (ecosystem) OR (bacteria) OR (flora) OR (microflora) OR (dysbiosis)) | 276,532 |
| 2 | “Sarcopenia” [Mesh] | 10,418 |
| 3 | #1 AND #2 | 124 |
| 4 | Limits: humans | 111 |
| 5 | Limits: English | 108 |
| **B** Embase Search | | |
| Entry | Embase Search Strategy | Results |
| 1 | gut | 226,705 |
| 2 | gastrointestinal | 871,306 |
| 3 | intestinal | 465,623 |
| 4 | fecal | 112,486 |
| 5 | stool | 78,588 |
| 6 | #1 OR #2 OR #3 OR #4 OR #5 | 1,412,962 |
| 7 | microbiome | 76,469 |
| 8 | microbiota | 104,148 |
| 9 | ecosystem | 153,508 |
| 10 | bacteria | 555,364 |
| 11 | flora | 160,284 |
| 12 | microflora | 73,950 |
| 13 | dysbiosis | 25,534 |
| 14 | #7 OR #8 OR #9 OR #10 OR #11 OR #12 OR #13 | 890,044 |
| 15 | #6 AND #14 | 161,969 |
| 16 | 'sarcopenia'/exp | 18,969 |
| 17 | #15 AND #16 | 228 |
| 18 | Limits: English | 211 |
| 19 | Limits: humans | 204 |
| **C** Cochrane Library Search | | |
| Entry | Cochrane Library Search Strategy | Results |
| 1 | ((gut) OR (gastrointestinal) OR (intestinal) OR (fecal) OR (stool)) AND ((microbiome) OR (microbiota) OR (ecosystem) OR (bacteria) OR (flora) OR (microflora) OR (dysbiosis)) | 13468 |
| 2 | “Sarcopenia” [Mesh] | 899 |
| 3 | #1 AND #2 | 26 |
