# Supplementary material for: Gut microbial composition is altered in sarcopenia: A systematic review and meta-analysis of clinical studies
Source: PLoS One. 2024 Aug 6;19(8):e0308360. doi: 10.1371/journal.pone.0308360 (PMC11302912; doi:10.1371/journal.pone.0308360)
Supplement: S3 Table — (DOCX) [file pone.0308360.s003.docx]

**S3 Table**. The Joanna Briggs Institute (JBI) critical appraisal checklist for analytical cross-sectional studies for assessing the quality of comparative studies in the meta-analysis.

| **Study** | **Were the criteria for inclusion in the sample clearly defined?** | **Were the study subjects and the setting described in detail?** | **Was the exposure measured in a valid and reliable way?** | **Were objective, standard criteria used for measurement of the condition?** | **Were the confounding factors identified?** | **Were strategies to deal with confounding factors stated?** | **Were the outcomes measured in a valid and reliable way?** | **Was appropriate statistical analysis used?** |
| --- | --- | --- | --- | --- | --- | --- | --- | --- |
| Picca 2019 | ★ | ★ | ★ | ★ | - | - | ★ | ★ |
| Ticinesi 2020 | ★ | ★ | ★ | ★ | ★ | ★ | ★ | ★ |
| Kang 2021 | ★ | ★ | ★ | ★ | - | - | ★ | ★ |
| Margiotta 2021 | ★ | ★ | ★ | ★ | - | - | ★ | ★ |
| Ponziani 2021 | ★ | ★ | ★ | ★ | ★ | ★ | ★ | ★ |
| Lee 2022 | ★ | ★ | ★ | ★ | - | - | ★ | ★ |
| Zhou 2022 | ★ | ★ | ★ | ★ | - | - | ★ | ★ |
| Wang 2022 | ★ | ★ | ★ | ★ | ★ | ★ | ★ | ★ |
| Wu 2022 | ★ | ★ | ★ | ★ | - | - | ★ | ★ |
| Han 2022 | ★ | ★ | ★ | ★ | - | - | ★ | ★ |
| Ni Lochlainn 2023 | ★ | ★ | ★ | ★ | ★ | - | ★ | ★ |
| Peng 2023 | ★ | ★ | ★ | ★ | - | - | ★ | ★ |
| Yang 2023 | ★ | ★ | ★ | ★ | ★ | ★ | ★ | ★ |
| Wang 2023 | ★ | ★ | ★ | ★ | ★ | - | ★ | ★ |
| Lee 2023 | ★ | ★ | ★ | ★ | - | - | ★ | ★ |
| Yan 2023 | ★ | ★ | ★ | ★ | ★ | - | ★ | ★ |
| Lou 2024 | ★ | ★ | ★ | - | - | - | ★ | ★ |
| Shan 2024 | ★ | ★ | ★ | ★ | - | - | ★ | ★ |
| Zhang 2024 | ★ | ★ | ★ | ★ | - | - | ★ | ★ |

Abbreviations: ★: yes; -: no/unclear

The confounding factor mentioned in the fifth question is mainly referred to the diet.
