# Supplementary material for: Gut microbial composition is altered in sarcopenia: A systematic review and meta-analysis of clinical studies
Source: PLoS One. 2024 Aug 6;19(8):e0308360. doi: 10.1371/journal.pone.0308360 (PMC11302912; doi:10.1371/journal.pone.0308360)
Supplement: S4 Table — (DOCX) [file pone.0308360.s004.docx]

##### S4 Table. GRADE evidence profile

| Certainty assessment | | | | | | | | | Summary of findings | | | Certainty |
| --- | --- | --- | --- | --- | --- | --- | --- | --- | --- | --- | --- | --- |
|  |  |  |  |  |  |  |  |  | Participants |  | Effect |  |
| No of studies | Design | Initial rating | Risk of bias | Inconsistency | Indirectness | Imprecision | Publication bias | Upgrading Domains | No of  participants |  | Absolute  (95 % CI) |  |
| **Chao1** | | | | | | | | | | | | |
| 12 | observational | Low | Not serious | Serious(-1)^1^ | Not serious | Not serious | Not serious | No upgrading | 1051 |  | SMD -0.44  (-0.64 to -0.23) | Very low certainty |
| **Observed species** | | | | | | | | | | | | |
| 5 | observational | Low | Not serious | Serious(-1)^1^ | Not serious | Not serious | Serious(-1)^3^ | No upgrading | 607 |  | SMD-0.68 (-1.00 to -0.37) | Very low certainty |
| **ACE index** | | | | | | | | | | | | |
| 4 | observational | Low | Not serious | Not serious | Not serious | Serious(-1)^2^ | Serious(-1)^3^ | No upgrading | 249 |  | SMD-0.30 (-0.56 to -0.04) | Very low certainty |
| **Index of species richness** | | | | | | | | | | | | |
| 1 | observational | Low | Not serious | NA | Not serious | Serious(-1)^2^ | Serious(-1)^3^ | No upgrading | 17 |  | SMD-0.13 (-1.13 to 0.86) | Very low certainty |
| **Shannon index** | | | | | | | | | | | | |
| 10 | observational | Low | Not serious | Not serious | Not serious | Not serious | Not serious | No upgrading | 4874 |  | SMD0.02 (-0.08 to 0.13) | Low certainty |
| **Simpson index** | | | | | | | | | | | | |
| 6 | observational | Low | Not serious | Not serious | Not serious | Serious(-1)^2^ | Serious(-1)^3^ | No upgrading | 371 |  | SMD0.00 (-0.20 to 0.20) | Very low certainty |
| **Phylogenetic diversity** | | | | | | | | | | | | |
| 1 | observational | Low | Not serious | NA | Not serious | Serious(-1)^2^ | Serious(-1)^3^ | No upgrading | 50 |  | SMD-0.56 (-1.12 to 0.01) | Very low certainty |

Explanation:

^1^:I-square ≥50%, downgraded with one level.

^2^: Fewer than 400 participants in the pooling, downgraded with one level.

^3^: Fewer than 10 studies have been pooled, making it impossible to conduct publication bias; downgraded with one level.

NA: not applicable.
