# Supplementary material for: Gut microbial composition is altered in sarcopenia: A systematic review and meta-analysis of clinical studies
Source: PLoS One. 2024 Aug 6;19(8):e0308360. doi: 10.1371/journal.pone.0308360 (PMC11302912; doi:10.1371/journal.pone.0308360)
Supplement: S5 Table — (DOCX) [file pone.0308360.s005.docx]

**S5 Table**. Subgroup analysis of comparison between sarcopenia and non-sarcopenia for richness in the gut microbiota.

|  |  | No. of | |  |  |
| --- | --- | --- | --- | --- | --- |
| Variable | No. of Trials | With sarcopenia | Total | Richness, SMD (95%CI) | *P* Value |
| Region |  |  |  |  | 0.34 |
| East | 10 | 405 | 916 | -0.45(-0.63 to -0.26) |  |
| West | 4 | 56 | 152 | -0.27(-0.60 to 0.06) |  |
| Definition of sarcopenia |  |  |  |  | 0.13 |
| AWGS2019 | 7 | 185 | 442 | -0.28(-0.54 to -0.02) |  |
| EWGSOP | 2 | 93 | 209 | -0.45(-0.73 to -0.18) |  |
| FNIH | 3 | 51 | 135 | -0.29(-0.64 to 0.06) |  |
| IWGS | 1 | 24 | 76 | -0.93(-0.56 to -0.25) |  |
| Research setting |  |  |  |  | 0.82 |
| Community dwellers | 6 | 122 | 297 | -0.38(-0.67 to -0.08) |  |
| Patients | 8 | 339 | 771 | -0.42(-0.60 to -0.23) |  |
| Age |  |  |  |  | 0.61 |
| ＜60 | 2 | 61 | 122 | -0.53(-1.04 to -0.02) |  |
| ≥60 | 12 | 400 | 946 | -0.39(-0.56 to -0.22) |  |
| Female (%) |  |  |  |  | 0.48 |
| ＜50 | 7 | 258 | 543 | -0.34(-0.59 to -0.09) |  |
| ≥50 | 7 | 203 | 525 | -0.45(-0.66 to -0.25) |  |
| BMI (kg/m^2^) |  |  |  |  | 0.19 |
| ＜24 | 6 | 213 | 523 | -0.53(-0.72 to -0.33) |  |
| ≥24 | 6 | 204 | 438 | -0.27(-0.60 to 0.06) |  |
| Method to measure gut microbiota |  |  |  |  | 0.59 |
| Shotgun metagenomic sequencing | 1 | 5 | 17 | -0.41(-0.57 to -0.25) |  |
| 16S rRNA sequencing of V3-V4 | 13 | 456 | 1051 | -0.13(-1.13 to 0.86) |  |
| Publication year |  |  |  |  | 0.54 |
| ＜2022 | 5 | 67 | 223 | -0.33(-0.62 to -0.03) |  |
| ≥2022 | 9 | 394 | 845 | -0.44(-0.63 to -0.25) |  |
| Sample size |  |  |  |  | 0.36 |
| ＜62 | 7 | 130 | 319 | -0.27(-0.60 to 0.06) |  |
| ≥62 | 7 | 331 | 749 | -0.45(-0.63 to -0.26) |  |
