# Supplementary material for: Gut microbial composition is altered in sarcopenia: A systematic review and meta-analysis of clinical studies
Source: PLoS One. 2024 Aug 6;19(8):e0308360. doi: 10.1371/journal.pone.0308360 (PMC11302912; doi:10.1371/journal.pone.0308360)
Supplement: S6 Table — (DOCX) [file pone.0308360.s006.docx]

**S6 Table**. Subgroup analysis of comparison between sarcopenia and non-sarcopenia for diversity in the gut microbiota.

|  |  | No. of | |  |  |
| --- | --- | --- | --- | --- | --- |
| Variable | No. of Trials | With sarcopenia | Total | Diversity, SMD (95%CI) | *P* Value |
| Region |  |  |  |  | 0.90 |
| East | 9 | 368 | 1,914 | 0.01(-0.12 to 0.15) |  |
| West | 1 | 129 | 2,991 | 0.00(-0.18 to 0.18) |  |
| Definition of sarcopenia |  |  |  |  | 0.10 |
| AWGS2019 | 8 | 344 | 1,838 | 0.06(-0.09 to 0.20) |  |
| EWGSOP | 1 | 129 | 2,991 | 0.00(-0.18 to 0.18) |  |
| IWGS | 1 | 24 | 76 | -0.49(-0.98 to -0.01) |  |
| Participants |  |  |  |  | 0.11 |
| Community dwellers | 6 | 369 | 4,653 | 0.04(-0.07 to 0.16) |  |
| Patients | 4 | 128 | 252 | -0.22(-0.52 to 0.08) |  |
| Age |  |  |  |  | 0.13 |
| ＜60 | 3 | 90 | 172 | -0.26(-0.64 to 0.11) |  |
| ≥60 | 6 | 390 | 4,686 | 0.03(-0.08 to 0.15) |  |
| Female (%) |  |  |  |  | 0.41 |
| ＜50 | 5 | 269 | 1,669 | 0.05(-0.10 to 0.20) |  |
| ≥50 | 5 | 228 | 3,236 | -0.04(-0.20 to 0.12) |  |
| BMI (kg/m^2^) |  |  |  |  | 0.53 |
| ＜24 | 5 | 255 | 1,677 | 0.03(-0.11 to 0.18) |  |
| ≥24 | 3 | 198 | 3,121 | -0.04(-0.21 to 0.13) |  |
| Method to measure gut microbiota |  |  |  |  | 0.05 |
| Shotgun metagenomic sequencing | 1 | 141 | 1417 | 0.14(-0.03 to 0.32) |  |
| 16S rRNA sequencing of V3-V4 | 9 | 356 | 3,488 | -0.08(-0.22 to 0.06) |  |
| Publication year |  |  |  |  | 0.33 |
| ＜2023 | 4 | 222 | 1613 | 0.06(-0.09 to 0.21) |  |
| ≥2023 | 6 | 275 | 3,344 | -0.05(-0.21 to 0.11) |  |
| Sample size |  |  |  |  | 0.35 |
| ＜60 | 4 | 103 | 217 | -0.12(-0.42 to 0.18) |  |
| ≥60 | 6 | 394 | 4,688 | 0.03(-0.09 to 0.15) |  |
