# Supplementary material for: Gut microbial composition is altered in sarcopenia: A systematic review and meta-analysis of clinical studies
Source: PLoS One. 2024 Aug 6;19(8):e0308360. doi: 10.1371/journal.pone.0308360 (PMC11302912; doi:10.1371/journal.pone.0308360)
Supplement: S2 Fig — A) Level: phylum; B) Level: family; C) Level: genus; D) Level: species. (PDF) [file pone.0308360.s008.pdf]

S2 Figure. The relative abundance of gut microbes between sarcopenia and non-sarcopenia in each study. A) Level: phylum; B) Level: family; C) Level: genus; D) Level: species.

A

	Firmicutes	Euryarchaeota	Actinobacteria	Verrucomicrobia	Proteobacteria	Bacteroidetes	Fusobacteria	Synergistetes
Kang 2021	↓							
Ponziani 2021	=	↓	=	=	↓	=		
Wu 2022	↑		=		↓	↑		
Wang 2023	↑		↑		↓	↓		
Lee 2023	=		↓	↑	↑	=	↑	=
Zhou 2022								
Yang2023			↑					
Peng 2023	↓		↑		↑	↓		↑
Lee 2022	=		=	=	=	=		
Picca 2019	=	=	=	=	=	=		=
shan2024	=		=		=	=		

↑ increased
↓ decreased
= no difference
not examined/ not reported

B

	Porphyromonadaceae	Prevotellaceae	Rikenellaceae	Tannerellaceae	Barnesiellaceae	Bacteroidaceae	Bifidobacteriaceae	Coriobacteriaceae	Eggerthellaceae	Micrococcaceae	Akkermansiaceae	Verrucomicrobiaceae	Erysipelotrichaceae	Ruminococcaceae	Lactobacillaceae
Kang 2021	↑													↑	
Ponziani 2021	=	↓	=		=		=	=		=		↓		=	=
Wu 2022			=											=	
Wang 2023															
Lee 2023		↓	=	↓	↑		↓	↓	↑	↑	↑		↓	↓	↑
Zhou 2022															
Yang2023	↑	↓	↑			↓	↑						↑		↑
Peng 2023		↓													
Margiotta 2021										↑		↑			
Picca 2019	=	=	=				↑	=				=	=	=	=

	Lachnospiraceae	Veillonellaceae	Peptostreptococcaceae	Streptococcaceae	Peptococcaceae	Leuconostocaceae	Enterococcaceae	Erysipelotrichaceae	Enterobacteriaceae	Pasteurellaceae	Enterobacteriaceae	Desulfovibrionaceae	Methanobacteriaceae	Fusobacteriaceae	Gemellaceae
Kang 2021	↓														
Ponziani 2021	=	=	=	=			=	=	=	=		=	↓		
Wu 2022	↓								=						
Wang 2023															
Lee 2023	↓	↓	↑	↑	↑	↑	↑		↑	↓	↑	↑		↑	
Zhou 2022															
Yang2023		↑	↓					↓						↓	
Peng 2023			↓												
Margiotta 2021		↓													↓
Picca 2019	=	=	↑	=			=			=	=	=	=		

C

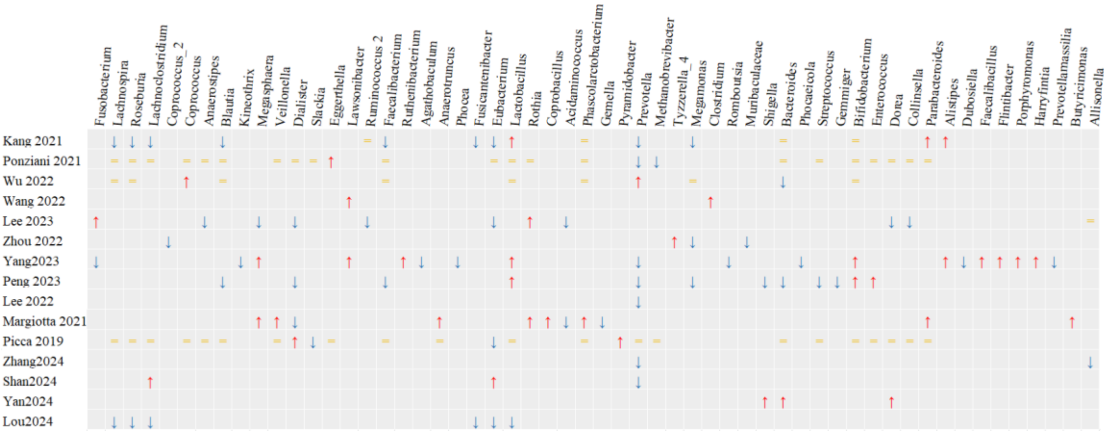
Fusobacterium
Lachnospira
Roseburia
Lachnoclostridium
Coprococcus_2
Coprococcus
Anaerostipes
Blautia
Kineothrix
Megasphaera
Veillonella
Dialister
Slackia
Eggerthella
Lawsonibacter
Ruminococcus 2
Faecalibacterium
Ruthenibacterium
Agathobaculum
Anaerotruncus
Phocea
Fusicantenibacter
Eubacterium
Lactobacillus
Rothia
Coprobacillus
Acidaminococcus
Phascolarctobacterium
Gemella
Pyramidobacter
Prevotella
Methanobrevibacter
Tyzzerella_4
Megamonas
Clostridium
Romboutsia
Muribaculaceae
Shigella
Bacteroides
Phocaeicola
Streptococcus
Gemmiger
Bifidobacterium
Enterococcus
Dorea
Collinsella
Parabacteroides
Alistipes
Dubosiella
Faecalibacillus
Flintibacter
Porphyromonas
Harryfintia
Prevotellamassilia
Butyricimonas
Allisonella
Kang 2021
Ponziani 2021
Wu 2022
Wang 2022
Lee 2023
Zhou 2022
Yang2023
Peng 2023
Lee 2022
Margiotta 2021
Picca 2019
Zhang2024
Shan2024
Yan2024
Lou2024

D

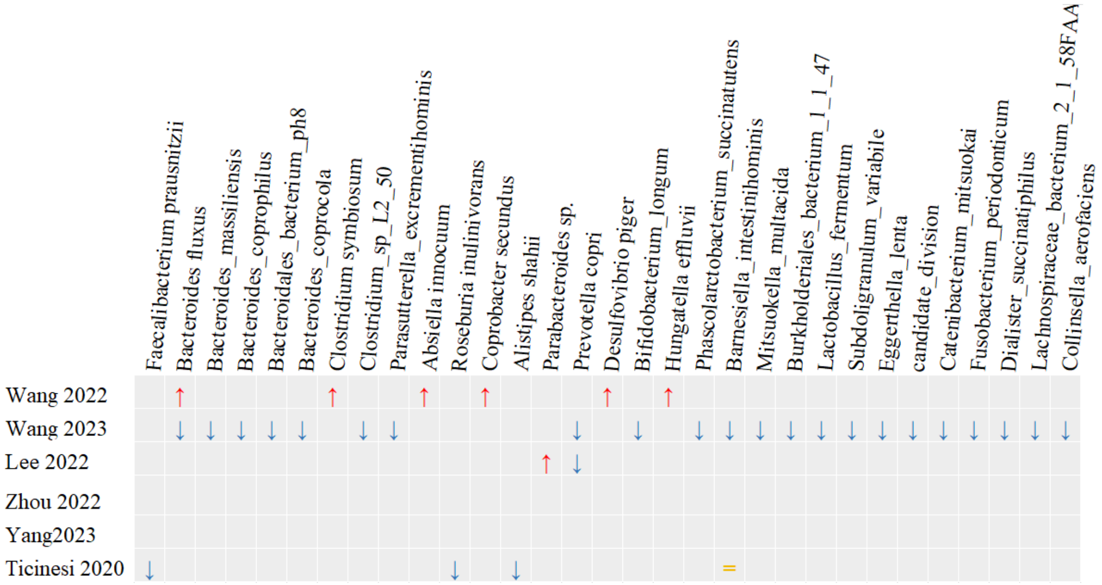
Faecalibacterium prausnitzii
Bacteroides fluxus
Bacteroides_massiliensis
Bacteroides_coprophilus
Bacteroidales_bacterium_ph8
Bacteroides_coprocola
Clostridium symbiosum
Clostridium_sp_L2_50
Parasutterella_excrementihominis
Absiella innocuum
Roseburia inulinivorans
Coprobacter secundus
Alistipes shahii
Parabacteroides sp.
Prevotella copri
Desulfovibrio piger
Bifidobacterium_longum
Hungatella effluvii
Phascolarctobacterium_succinatutens
Barnesiella_intestinihominis
Mitsuokella_multacida
Burkholderiales_bacterium_1_1_47
Lactobacillus_fermentum
Subdoligranulum_variabile
Eggerthella_lenta
candidate_division
Catenibacterium_mitsuokai
Fusobacterium_periodonticum
Dialister_succinatiphilus
Lachnospiraceae_bacterium_2_1_58FAA
Collinsella_aerofaciens
Wang 2022
Wang 2023
Lee 2022
Zhou 2022
Yang2023
Ticinesi 2020
